# Crosstalk between KIF15 and AR in castrate-resistant prostate cancers

**DOI:** 10.18632/oncoscience.556

**Published:** 2022-04-22

**Authors:** Hanny Yang, Xuesen Dong

**Keywords:** castrate-resisitant prostate cancer, adnrogen receptor, kinesin, KIF15, metosis

To develop therapy resistance to androgen receptor (AR) pathway inhibitors such enzalutamide (ENZ), prostate cancer (PCa) cells commonly adopt two surviving strategies. One is to strengthen the AR signaling to overcome the inhibitory effects of ENZ. It is frequently observed that AR gene amplification, overexpression, and generation of AR splicing variants occur in ~70–80% of castrate-resistant prostate cancers (CRPC) [1]. The other is to bypass the dependence of the AR signaling for survival and progress to AR indifferent neuroendocrine tumors or double negative tumors. While persistent AR protein expression in CRPC patient biopsies is observed in agreement with AR mRNA overexpression, the dilemma is that antagonist bound AR is more quickly degraded than agonist bound AR. Therefore, it is reasonable to speculate that persistent AR expression in ENZ-resistant (ENZ-R) tumors must possess mechanisms to prevent AR proteins from degradation.

Recently, Gao et al., [2] reported that a mitosis-relevant kinesin family, KIF15, enhances AR and AR-V7 protein stability in ENZ-R cell and xenograft models. First, they showed upregulation of KIF15 in several CRPC patient samples as well as ENZ-R cell and xenograft models. KIF15 silencing re-sensitizes ENZ-R cells to ENZ treatment. Second, they showed that KIF15 forms a protein complex with AR and USP14. While USP14 is an enzyme that can remove poly-ubiquitination modifications of AR, the recruitment of USP14 by KIF15 prevents AR protein from degradation through the ubiquitin-proteasome pathway. Third, they demonstrated that KIF15 is an AR target gene, whereby transcriptionally active AR can be recruited to KIF15 promoters to stimulate KIF15 gene transcription. Last, they showed combining ENZ and Kif15-IN-1 (a KIF15 inhibitor) resulted in stronger tumor suppression. Intriguingly, parallel research by the same group also showed that KIF15 enhances EGFR signaling to contribute to CRPC progression [3]. Together, these findings reveal a critical molecular mechanism by which KIF15 promotes ENZ-R progression and may be served as a drug target for ENZ-R PCa.

Kinesins [4] are a superfamily of ATP-dependent motor proteins responsible for the directional transport of organelles, proteins, and mRNAs intracellularly. In particular, KIF4 and KIF10 family members are chromokinesins, which have DNA-binding domains to exert non-redundant mitotic functions such as chromosome alignment, central spindle formation, and polar ejection force generation during mitosis. KIF4 members are exclusively bound to chromosomes until metaphase and relocate to the spindle midzone during anaphase with their main functions relating to the mitotic spindle fibers. In contrast, KIF15 is different from other kinesin members in that it is localized in the cytoplasm and does not interact with microtubules during interphase. At the metaphase and anaphase, KIF15 interacts with TPX2 and Ki67 proteins to be associated with microtubules and chromosomes. It plays an important role in bipolar spindle formation and spindle pole separation. However, the function of KIF15 during interphase remains largely unknown. The novel findings from Gao’s studies [2, 3] are that they revealed that KIF15 enhances the AR and EGFR signaling in the cytoplasm to contribute to ENZ-R PCa progression.

The question remains as to whether enhanced KIF15 expression is responsible for ENZ-resistance of PCa or it is a consequence of ENZ-resistance. The authors provided convincing evidence that the gain-of-function of KIF15 conferred PCa cells growth advantage under ENZ treatment. However, they also showed that agonist-activated AR was recruited to the KIF15 promoter to upregulate its transcription. Prolonged ENZ treatment also induced KIF15 expression. These results suggest that re-activated AR signaling in ENZ-R cancer cells is responsible for KIF15 upregulation. However, increased KIF15 expression may also reflect the proliferative status of ENZ-R cells since these cells were selected by ENZ treatment with gained proliferative phenotypes, and KIF15 is known for its essential role during mitosis. KIF15 maybe be a multifunctional protein that may be a part of the complicated mechanisms contributing to ENZ-R and is also needed for mitosis of ENZ-R cells driven by AR and AR splice variants.

It would be interesting to study the temporal and spatial relationship between KIF15 and AR. The AR protein is shuffling between the cytoplasm and nucleus during interphase, while liganded-AR is closely bound to the mitotic chromatin during all the stages [5]. AR uses its ligand-binding domain to interact with the microtubule in the absence of androgens but is disassociated from the microtubule in the presence of androgens and becomes a cargo of dynein to be shipped to the nucleus along the microtubule [6]. This explains why taxanes acts to stabilize the microtubule and also blocks AR protein to be localized to the nucleus. KIF15 on the other side is mainly localized in the cytoplasm and does not bind microtubules. It binds to the spindle microtubule during metaphase and anaphase [7]. It would be interesting to study where and how the protein complex of KIF15, AR, and USP14 is formed throughout cell cycling in hormone-sensitive and ENZ-R PCa cells.

The molecular mechanism by which co-targeting KIF15 and AR to achieve stronger tumor suppression needs further investigation. Kif15-IN-1 is an inhibitor of KIF15 [8] and its tumor suppression effects are more likely attributed to its destructive effects on cancer cell mitosis. However, the authors also showed that Kif15-IN-1 enhances AR and AR-V7 protein degradation. Given that the mode of action of Kif-IN-1 in suppressing KIF15 had not been fully characterized, it remains unclear whether Kif15-IN-1 enhances AR protein degradation either through inhibiting KIF15 kinase activity or by disrupting the KIF15/AR/USP14 protein complex.

In summary, this study provides a novel mechanism by which the mitotic kinesin KIF15 increases AR protein stability and propose a combination therapy of co-targeting KIF15 and AR to improve PCa patient outcome.
